# Effect of treadmill ambulatory training on glucose control and blood pressure in persons with type 2 diabetes: A pilot study

**DOI:** 10.1371/journal.pone.0298179

**Published:** 2024-04-04

**Authors:** Ulric Sena Abonie, Raphael Aseye Addo, Laureen Kumah, Ama Kissiwaa Ofori – Ampomah, Vincent Makinyi

**Affiliations:** 1 Department of Sport, Exercise & Rehabilitation, Northumbria University, Coach Lane Campus, Benton, Newcastle upon Tyne, United Kingdom; 2 Department of Physiotherapy and Rehabilitation Sciences, University of Health and Allied Sciences, Ho, Volta Region-Ghana; University of Rwanda College of Medicine and Health Sciences, RWANDA

## Abstract

**Background:**

Lack of time is often cited by persons with type 2 diabetes for non-participation in regular exercise. This highlights the need to explores ways to help persons with type 2 diabetes to engage in an active lifestyle. This study evaluated the effect of a short duration norm intensity exercise on blood glucose and blood pressure in persons with type 2 diabetes.

**Methods:**

Twenty persons with type 2 diabetes were randomly assigned to either training group (n = 10) or control group (n = 10). The training group received 4-weeks ambulatory training on a motor-driven treadmill (2 x 20 min per week at 60% target heart rate). The control group received no training. Blood glucose, and systolic and diastolic blood pressures were assessed before and after the 4-weeks training. Repeated measures ANOVA were used to examine training effect.

**Results:**

Training significantly improved blood glucose (mean difference = -2.73; p = 0.03). No effects were found for systolic blood pressure (mean difference = -0.30; p = 0.96) and diastolic blood pressure (mean difference = -0.90; p = 0.82).

**Conclusion:**

Training improved blood glucose but not blood pressure. A short-duration ambulatory training is an appropriate exercise mode to elicit beneficial effect, and exercise adoption in persons with type 2 diabetes.

**Trial registration:**

This pilot trial is registered with the Pan African Clinical Trial Registry at pactr.samrc.ac.za (PACTR202306601940612).

## Introduction

Type 2 diabetes is a global health challenge, with about 80% of type 2 diabetes cases recorded in developing countries [1]. In Africa, there are 19.4 million adults between the ages of 20–79 years living with diabetes [2]. Specifically, in Ghana, there is a significant type 2 diabetes burden in terms of its costly treatment and associated premature morbidity and mortality [3, 4]. The prevalence of diabetes is increasing and has been associated with increasing age, sedentary lifestyle, growing urbanization cultures, processed diets, hypertension, alcohol consumers and obesity [5–7]. Given the prevalence of type 2 diabetes in developing countries [1–7], population-based efforts to reduce its burden are as critical [8].

Hypertension and uncontrolled blood glucose are common features seen in persons with type 2 diabetes [9, 10]. Hypertension is the leading cause of mortality and morbidity in persons with diabetes, and the leading cause of the direct and indirect costs associated with type 2 diabetes [9]. Conversely, uncontrolled blood glucose predisposes persons with type 2 diabetes to impaired cognitive function, and cardiovascular and diabetes induced complications [10]. Consequently, treatment goals for persons with type 2 diabetes includes achieving and maintaining optimal blood glucose and blood pressure to prevent or delay the progression of chronic complications [11, 12].

Studies have shown that exercise has health-enhancing impact such as positive effects on blood pressure, cardiovascular risk factor, quality of life, glycemic control, mobility and participation in daily life and cognitive function [13–16]. However, participation in regular physical activity is low among persons with type 2 diabetes in comparation to those without the condition [17, 18]. It is recommended that persons with type 2 diabetes engage in at least 150 minutes of moderate-intensity (50–70% of maximum heart rate) aerobic exercise each week, with a two-day rest period between exercises [13, 19]. This time commitment is in addition to all the other self-care activities recommended for persons with diabetes. Consequently, lack of time is an often-cited reason for non-participation in exercise, by persons with type 2 diabetes [20]. Thus, the recommended 150 minutes of exercise per week may not be practical, appealing and attainable.

A short duration norm intensity ambulatory exercise could be central to the adoption and maintenance of physical activity behaviour and can provide the platform to engage in graduated activity or exercises. Exercising for a shorter duration would be perceived as achievable and can improve motivation to remain active. This could be particularly beneficial for persons with type 2 diabetes who are non-exercisers, physically inactive or for whom lack of time is a barrier to engaging in an active lifestyle. However, the potential of a short duration norm intensity ambulatory exercise to improve blood glucose and blood pressure in persons with type 2 diabetes have not been explored. Therefore, the aim of this study was to investigate whether a 20-minute moderate intensity (60% target heart rate) ambulatory treadmill exercise twice weekly could improve blood glucose and blood pressure in persons with type 2 diabetes. We hypothesized that the ambulation exercise would improve blood glucose and blood pressure.

## Materials and method

### Participants

Community dwelling adults were recruited from the Diabetes Clinic of the Ho Teaching Hospital, Ghana which provides medical care for people with diabetes through advertisement. All study procedures were approved by the Research Ethics Committee of the University of Health and Allied Sciences (reference: UHAS-REC A.8(21)2021) and were conducted in accordance with the Declaration of Helsinki. Those interested in participation were contacted by the researchers who explained the study rationale, potential benefits, procedures, and answered all questions. Interested participants were screened with the Physical Activity Readiness Questionnaire (PAR-Q) [21] to ensure they had no contraindications to participate in the study. Criteria for inclusion were persons diagnosed with type 2 diabetes for at least a year, aged 18 years and older, not currently or recently (in the previous 12 months) engaged in a physical activity program, ambulatory (with or without assistive device) and consistent dietary pattern for at least 6 months. Participants were excluded from the study if they were not able to complete the questionnaire, had recently started a new treatment including medication or changed treatment, had comorbid conditions that may influence understanding, or answering of the questionnaires. Informed written consent was obtained from eligible participants. Participants were asked to inform researchers of change or initiation of medical or conservative treatment during the study period. A priori sample size calculation was performed using GPower (v3.0.0) using the effect size from Mendes et al., [22] (ηp^2^ = 0.52) that demonstrates blood glucose reduction following treadmill walking. To detect the specified effect, an estimated sample size of 6 participants per group is required. To maximise statistical power and account for potential drop out, a minimum of 10 participants per group were recruited.

### Procedure

Recruitment for this study began in April 2021 and ended in July, 2021. The main data collection periods were at the baseline and the 4-weeks follow-up. At baseline, demographic and health status information were collected. Background demographics included age, gender, diabetes diagnosis, medication use and body mass index, blood glucose and blood pressure. Participants were then stratified by age and gender and randomized into training (n = 10) or control group (n = 10) by RAA and LK. Participants blindly picked a folded paper marked with “training” or “control” out of a box. The training began within the week after the baseline assessment. At 4-weeks follow-up, the blood glucose level and blood pressure of all participants were assessed.

Participants in the training group familiarized with the training set-up, two 5-min familiarization trials on a motor driven treadmill (Enraf Nonius, The Netherlands). After the familiarization trials, participants performed a submaximal test to determine the required velocity which elicited an intensity of 60% Target Heart Rate (THR). The initial velocity was 1m·s^−1^ and increased by 0.5m·s^−1^ every 30sec until an intensity of 60% THR was elicited. The test was terminated after 1min at the 60% THR.

The training group received 4-weeks of short duration ambulatory training on a motor driven treadmill, 2 x 20 min/week at 60% THR; details provided in the next section. The control group did not receive any training. Post-tests were conducted at the same time of the day, and on the same day of the week, four weeks after the pre-tests were completed. All participants were asked to maintain their regular daily physical activity pattern and diet during the study period. The control group was given the opportunity to partake in the training after the study.

### Training

All training sessions were performed on a motor-driven treadmill (Enraf Nonius, The Netherlands) in a climate-controlled physiotherapy gymnasium. The training group received 20 minutes ambulation training, two times a week for 4-weeks. The training sessions were performed at 60% THR, determined conform, as described in Karvonen et al., [23].

Resting heart rate (HR_rest_) was measured before training (Polar Accurex Plus; Polar Electro, OY, Finland) to calculate THR. To measure HR_rest_, participants sat quietly for 10 min, before commencement of the warm-up preceding the training. The final minute was used as HR_rest_. THR was calculated using the formula:

$${\text{HR}}_{\text{max}}=220–\text{age}$$ (1)

$$\text{Target}\;\text{Heart}\;\text{Rate}\;\left(\text{THR}\right)=\left[\left({\text{HR}}_{\text{max}}–{\text{HR}}_{\text{rest}}\right)\times \%\;\text{Intensity}\right]+{\text{HR}}_{\text{rest}}$$ (2)

During the training, velocity was increased when necessary (as fitness improved), to reach mean exercise intensity of 60% THR. Training sessions were monitored by heart rate (Polar Electro, Finland). During the last three minutes of each training session HR were obtained. Participants in the training group completed 8 training sessions.

At the start of each training session, participants performed a 5-min warm-up on a rowing ergometer. After the training session, a 5-min cool-down on a rowing ergometer was performed. A minimum of 2-day rest period was allowed in between training sessions.

### Outcomes

#### Blood glucose

Blood glucose was measured with a clinically validated digital and automatic glucometer (Breeze 2, Bayer Healthcare, Mishawaka, USA) [24] via a finger-prick test with a specific device (Microlet 2, Bayer Healthcare, Mishawaka, USA) by trained nurses. Glucometer calibration was tested against a standard solution before each test. Participants fasted (only water was permitted) for a minimum period of 8 hours prior to the test.

#### Blood pressure

Blood pressure was measured by researchers in the left upper arm using an appropriately sized cuff and a clinically validated digital and automatic blood pressure monitor (M6 Comfort, Omron Healthcare, Kyoto, Japan) [25] according to international recommendations [26]. Where possible, the same researcher obtained the blood pressure in each participant. After 5 minutes of sitting at rest, blood pressure was measured twice, with an interval of 5 minutes between readings. If the blood pressure readings differed by more than 5 mmHg, additional readings were obtained. The mean of two consecutive readings within 5 mmHg of each other was used as the value for that visit.

### Data analysis

All statistical analyses were performed using version 26.0 of the IBM statistical package for the social sciences software. All values were reported using descriptive statistics of mean (M), +- standard deviation (SD) to summarise the characteristics of participants [27]. An independent t-test was used to determine the baseline differences between groups. The effect of the training on blood glucose and blood pressure was evaluated with a 2 factor repeated measures ANOVA. The difference between baseline (pre-test) and 4-weeks follow-up (post-test) was used as within-subject factor and group as between-subjects factor. Effect size was calculated using partial eta-squared (ηp^2^) and interpreted as small (≥0.01), medium (≥0.06), or large (≥0.14) [28]. The significance level was set at p ≤ 0.05 for all tests. Normality of data was checked using the Shapiro-Wilk test and showed data were generally normally distributed.

## Results

### Participant flow and characteristics

**Fig 1:**
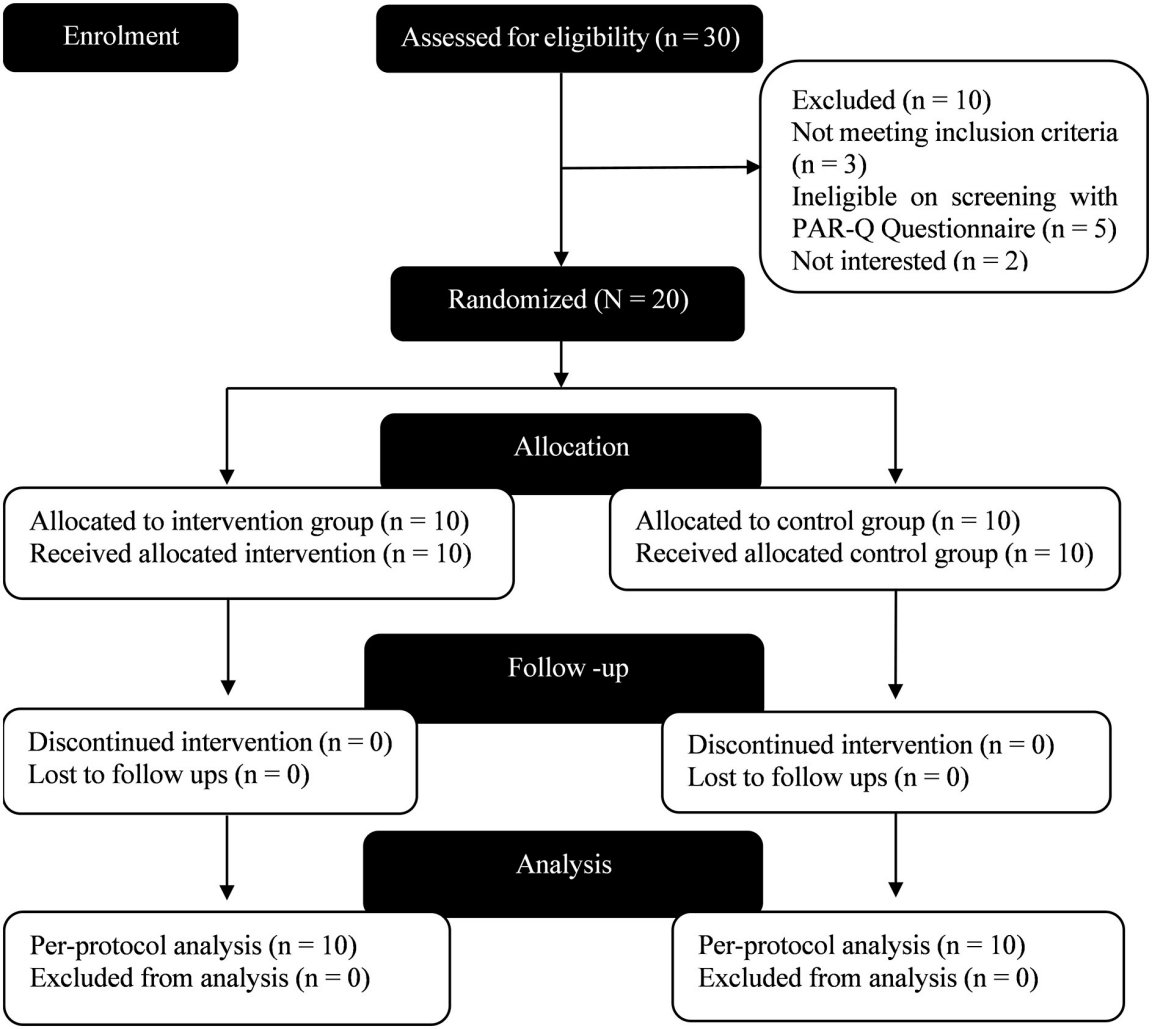
Flow diagram of participants through the study.

The flow chart of participants through the study and reasons for exclusion and withdrawal are displayed in Fig 1.

Of the 30 individuals who were assessed for the study eligibility, 20 were identified as eligible and were randomly assigned to either the training or the control group (training group: n = 10, control group: n = 10). Ten were excluded from the study (three of the participants failed to meet the inclusion criteria, five individuals ruled out of the study after screening with the PAR-Q questionnaire, and two persons withdrew their interest in the study). Demographics and baseline measures of the participants are presented in Table 1.

**Table 1:** Demographic characteristics of participants.

Variable		Training Group	Control Group	p
Number of participants		10	10	
Age, years (M ± SD)		50.60 ± 8.04	45.30 ± 7.06	0.14
Gender	Males (%)	3 (30)	4 (40)	1
	Females (%)	7 (70)	6 (60)	1
Body mass index, kg/m^2^ (M ± SD)		25.02 ± 0.87	25.46 ± 0.75	0.24
Blood Glucose, mmol/l (M ± SD)		12.45 ± 3.84	14.54 ± 1.86	0.15
Systolic BP, mmHg (M ± SD)		127.10 ± 9.50	139.20 ± 12.56	0.03
Diastolic BP, mmHg (M ± SD)		72.80 ± 5.88	82.20 ± 7.69	0.21

M: Mean, SD: Standard Deviation, BP: Blood Pressure, p: p value

The study sample (n = 20) was 65% females and mean age was 47.95 ± 7.85 years. There were no significant differences between groups at the baseline, except for systolic blood pressure (127.10 ± 9.50 versus 139.20 ± 12.56, p = 0.03). Although not statistically significant, compared with the control group, participants in the training group were slightly older (50.60 ± 8.04 versus 45.30 ± 7.06 years, p = .14). Body mass index, blood glucose and diastolic blood pressure were similar in training and control groups: 25.02 ± 0.87 versus 25.46 ± 0.75 p = 0.24; 12.45 ± 3.83 versus 14.54 ± 1.86, p = 0.14; 78.10 ± 6.40 versus 82.20 ± 7.69 p = .21, respectively.

### Training session

Table 2 shows the average training velocity and intensity. The average velocity during training was 5.13 ± 0.56m·s^−1^ (range, 4.56–5.81m·s^−1^). The average training heart rate was 100.58 ± 3.22bpm (range, 95.30–104.29bpm).

**Table 2:** Training parameters.

	N	Minimum	Maximum	Mean	Std. Deviation
Velocity (m·s^−1^)	10	4.56	5.81	5.13	0.56
Target heart rate (bpm)	10	95.30	104.29	100.58	3.22

N: Number of participants, bpm: beats per minute, Std. Deviation: Standard Deviation

Influence of treadmill ambulatory training

### Influence of ambulatory treadmill training

Comparisons of blood glucose, systolic blood pressure and diastolic blood pressure before and after the training period are presented in Table 3.

**Table 3:** Changes in outcomes between baseline and follow up.

Outcome	Group	Pre-test	Post-test	Pre-post change	Mean^a^ difference
Blood glucose (mmol/l)	TG (n = 10)	12.45 ± 3.84	6.53 ± 1.42	-5.92 ± 2.76*	-2.73*
	CG (n = 10)	14.54 ± 1.86	11.35 ± 1.79	-3.19 ± 2.51*	
Systolic BP (mmHg)	TG (n = 10)	127.10±9.50	119.70 ± 9.12	-7.40 ± 11.35	-0.30
	CG (n = 10)	139.20 ± 12.56	132.10 ± 13.57	-7.10 ± 15.88	
Diastolic BP (mmHg)	TG (n = 10)	78.10±6.40	72.80 ± 5.88	-5.30 ± 8.73	-0.90
	CG (n = 10)	82.20 ± 7.69	77.80 ± 10.39	-4.40 ± 8.83	

Values are presented as mean ± standard deviation;

^a^ = pre-post changes between groups; BP = blood pressure; CG = control group; TG = training group;

*Significant difference from pre-test (p < 0.05)

### Blood glucose

Significant group x time interaction was found, showing a decrease in blood glucose (mmol/l) (Mean difference = 3.46; 95%CI (-5.36−-1.55); p = 0.03; ηp^2^ = 0.23) with greater decreases occurring in the TG. Additionally, significant main effect of time (decrease) was found in blood glucose (mmol/l) (Mean difference = -4.55; 95% CI (-5.80–3.32); p < 0.001; ηp^2^ = 0.77).

### Blood pressure

No significant group x time interactions were found for systolic blood pressure (Mean difference = -7.25; 95% CI (-13.54–1.77); p = 0.96; ηp^2^ = 0.00) and diastolic blood pressure (Mean difference = -4.55; 95% CI (-10.59–1.49); p = 0.82; ηp^2^ = 0.00). However, significant main effects of time were found for systolic blood pressure (Mean difference = -12.25; 95% CI (-13.74 –-0.76); p = .03; ηp^2^ = 0.24) and diastolic blood pressure (Mean difference = -4.85; 95% (-8.98 –-0.72) p = 0.02; ηp^2^ = 0.25).

## Discussion

This study examined the effectiveness of a 4-weeks ambulatory treadmill training (60% THR intensity) on blood glucose and blood pressure in persons with type 2 diabetes from a tertiary hospital in Ghana. The most striking outcome of the present study was improvement in blood glucose. This provides insight into the use of ambulatory training to improve blood glucose, providing further knowledge to use in the management of diabetes. No improvement in systolic and diastolic blood pressures were found. The study findings were unlikely to be biased by the fluctuating nature of the health status of persons with type 2 diabetes.

Aerobic exercise increases glucose transporter carrier proteins abundance and translocation from an intracellular compartment to the plasma membrane and transverse tubules, and hence blood glucose uptake by a pathway that is not dependent on insulin [13]. Exercise reduces blood glucose through an increase of the translocation of glucose transporter carrier proteins to the surface of muscle cells [29, 30]. In other words, exercise helps to improve diabetic status, insulin sensitivity and lipid profile, and reduce metabolic risk factors.

For blood glucose measured with glucose meter, a significant change and a large effect size was observed. We observed a larger decrease in the blood glucose of the training group (-5.92 ± 2.76) in comparison to the control group (-3.19 ± 2.51), indicating that the training had a significant effect on blood glucose. Our findings are in accordance with studies on exercise in persons with diabetes that showed reduction in blood glucose level, following moderate continuous physical exercise [22, 31, 32] but contrary to the study by Aggarwala et al., [12] which examined the effect of aerobic exercise on blood glucose level and found no beneficial effect. A possible explanation for the reduction in blood glucose may be the increase in utilization of blood glucose with regards to insulin response to exercise [22]. The large effect size obtained in this study points at the important role of short duration ambulatory training in persons with diabetes.

For blood pressure measured with sphygmomanometer, no significant training effects were found for systolic and diastolic blood pressures, indicating that the training had no effect on blood pressure. The findings of non-beneficial effects on systolic and diastolic blood pressures were similar to that reported by Dobrosielski et al., [31] and Loimaala et al., [33]. However, the studies by Dobrosielski et al., [31] and Loimaala et al., [33] used concurrent resistance and aerobic exercises.

Current physical activity recommendations for type 2 diabetes treatment suggests a weekly accumulation of a minimum of 150 minutes of moderate intensity (50–70% of maximum heart rate) aerobic exercise per week spread over at least three days per week with no more than 2 consecutive days without exercise [13, 19]. Many studies have demonstrated the effect of exercise on the reduction and consequent control of blood glucose in persons with type 2 diabetes. Most of these studies used intensity ≥ 70 maximum heart rate and duration ≥ 30 minutes [34]. However, this may not be practical and appealing, creating an unattainable target for people with type 2 diabetes. It is evident that persons with type 2 diabetes are less active compared to persons without diabetes and a lack of time is often cited as a reason for not exercising [19]. This highlights the need for achievable and reproducible targets to motivate persons with type 2 diabetes to engage in exercise, with time constraint usually cited as a barrier to engagement in an active lifestlye.

The large beneficial effect size coupled with the statistically significant reduction in blood glucose found in this current study has clinical significance and provides valuable insights. This indicates the effectiveness of low duration ambulatory treadmill intervention to improve blood glucose in persons with type 2 diabetes. Blood glucose reduction helps prevent or delay long-term complications in persons with type 2 diabetes. This intervention design may need to be incorporated in the management of persons with type 2 diabetes to help them control blood glucose which is important in promoting health in persons with diabetes. The low duration mode makes it practically acceptable for person with type 2 diabetes for whom time constraint is a significant barrier to engagement in a physically active lifestyle.

The small sample size in this pilot study limits our ability to generalise the study findings. Also, the study inclusion criterion of only ambulatory persons may have excluded people who were severely affected by type 2 diabetes (i.e., mostly wheelchair dependent or bedridden) from participating. Including these persons in future research is warranted. Conversely, the difference in systolic blood pressure at baseline might have affected the results. Furthermore, additional training sessions would be required to provide a comprehensive programme, and longer training weeks will provide more insight into the benefits of ambulatory treadmill training to persons with diabetes. Replicating the study in a larger sample is warranted to examine variables that may moderate the effects of the ambulatory treadmill intervention and consequently allow firm conclusions to be drawn.

## Conclusion

The current study showed that short duration norm intensity treadmill ambulation exercise has large beneficial effect on blood glucose in persons with type 2 diabetes. This preliminary finding is very promising and shows the beneficial effect of short duration treadmill ambulatory exercise on blood glucose for persons with type 2 diabetes. This could be particularly beneficial for persons with type 2 diabetes who are non-exercisers, physically inactive or for whom lack of time is barrier to engaging an active lifestyle. The study findings call for a larger study, including a follow up to evaluate the medium-term effect of the intervention. This short duration treadmill ambulatory intervention looks promising for the management of diabetes and provide the basis for incorporating short duration treadmill ambulatory exercise in the management of diabetes to control and improve blood glucose.

## Supporting information

S1 Checklist Completed CONSORT checklist. (DOCX)

S1 Questionnaire Inclusivity in global research questionnaire. (DOCX)
